# Morphological Changes of the Pituitary Gland in Patients with Irritable Bowel Syndrome Using Magnetic Resonance Imaging

**DOI:** 10.3390/jimaging10090226

**Published:** 2024-09-13

**Authors:** Jessica Abou Chaaya, Jennifer Abou Chaaya, Batoul Jaafar, Lea Saab, Jad Abou Chaaya, Elie Al Ahmar, Elias Estephan

**Affiliations:** 1 Neuroscience Research Center, Faculty of Medical Sciences, Lebanese University, Beirut, Lebanon; jessica.abouchaaya@hotmail.com; 2 Division of Endocrinology and Metabolism, Department of Internal Medicine, American University of Beirut Medical Center, Beirut, Lebanon; batoul.y.jaafar@gmail.com; 3 Department of Nutrition and Dietetics, Faculty of Public Health Section 2, Lebanese University, Beirut, Lebanon; jennifer.abouchaaya@outlook.com; 4 Faculty of Engineering, Sagesse University, Furn El Chebbak, Baabda, Lebanon; lea.saab@uls.edu.lb; 5 Lab-STICC, UMR 6285 CNRS, ENIB, 29200 Brest, France; jad.abouchaaya@ieee.org; 6 Applied Physics Lab, Faculty of Science, Lebanese University, Campus Fanar, Jdeideh, Lebanon; 7 Bioengineering Nanoscience Laboratory UR_UM104 (LBN), Montpellier University, 545 Avenue Prof. Viala, 34193 Montpellier Cedex, France

**Keywords:** irritable bowel syndrome, diagnosis, volume calculation, pituitary, gastrointestinal tract

## Abstract

Irritable bowel syndrome (IBS) is a gastrointestinal functional disorder characterized by unclear underlying mechanisms. Several theories propose that hyperactivation of the hypothalamic–pituitary–adrenal (HPA) axis leads to elevated cortisol levels and increased sensitivity of gut wall receptors. Given the absence of prior literature on this topic, our study aimed to investigate the potential for diagnosing IBS based on morphological changes in the pituitary gland, specifically its volume and grayscale intensity. Additionally, we aimed to assess whether factors such as gender, age, and body mass index influence these parameters. This retrospective study involved 60 patients, examining the volume and grayscale characteristics of their pituitary glands in the presence of IBS. Our findings revealed a positive correlation between pituitary gland volume and IBS diagnosis, although no significant correlation was observed with grayscale intensity. Due to the limited existing research and the small sample size of our study, further investigation with a larger cohort is warranted to validate these results.

## 1. Introduction

Irritable bowel syndrome (IBS) is a chronic functional gastrointestinal disorder affecting a substantial global population. It ranks as the most prevalent functional GI disorder, with estimates indicating a global prevalence of approximately 11%. IBS is characterized by a constellation of symptoms including chronic abdominal pain, urgency, bloating, and altered bowel habits [1]. Diagnosis primarily relies on symptom-based criteria as outlined in the Rome IV criteria, necessitating the exclusion of other gastrointestinal disorders such as inflammatory bowel disease [2]. Studies in North America suggest a prevalence range of 10% to 15% in the general population, although many affected individuals do not seek medical attention [3]. It exhibits a higher prevalence among females and older adults aged 50 years and above compared to males [4]. While IBS itself is not life-threatening, it significantly diminishes the quality of life for those affected, impacting aspects such as education, employment, and social interactions [5]. Moreover, it is associated with increased healthcare expenditures and accounts for a substantial proportion of gastroenterology referrals in the United States, ranging from 25% to 50% [6]. IBS is also linked with several comorbid conditions including fibromyalgia, chronic fatigue syndrome, gastroesophageal reflux disease, functional dyspepsia, and psychiatric disorders [7,8]. Treatment approaches for IBS are multifaceted and may involve dietary modifications, pharmacological interventions, and non-pharmacological therapies such as cognitive–behavioral therapy and relaxation techniques [1].

The pathophysiology of irritable bowel syndrome (IBS) is intricate and not fully elucidated. Known risk factors include genetics, diet, and the gut microbiome, though their relative significance may vary across different regions influenced by geographical and cultural factors [1]. Symptoms are believed to arise from various factors such as visceral hypersensitivity, altered intestinal motility, neurotransmitter imbalances, infections, and psychosocial factors. Researchers have explored three main theories to explain the pathophysiology of IBS. One theory posits a hypersensitivity of gut wall receptors to specific stimuli, transmitting signals via afferent neural pathways to the brain [9]. Another theory suggests an elevated inflammatory status, supported by higher levels of cytokines observed in patients with IBS [10]. Our study focuses on the third theory, which proposes an overactivation of the hypothalamic–pituitary–adrenal (HPA) axis. This theory is substantiated by elevated cortisol levels resulting from increased adrenocorticotropin hormone (ACTH) release by the pituitary gland in individuals with IBS [11]. Further investigations into this theory include dynamic tests such as corticotropin-releasing hormone (CRH) stimulation, the dexamethasone challenge test, and the trier-social stress-test (TSST) [12,13,14,15,16].

The pituitary gland resides at the base of the human skull, housed within the sella turcica of the sphenoid bone, and comprises anterior and posterior lobes. In adults, the normal size of the pituitary gland measured by magnetic resonance imaging (MRI) ranges from 8 to 12 mm [17]. MRI is the gold standard for imaging sellar and parasellar regions due to its superior soft-tissue contrast, multiplanar capability, and avoidance of ionizing radiation. It provides detailed anatomical insights and assists in formulating medical or surgical management strategies [18]. The pituitary gland synthesizes various hormones crucial for regulating bodily functions such as growth, development, and stress response. Among these, the anterior lobe produces ACTH, essential for stimulating cortisol secretion from the adrenal glands. This interaction, known as the hypothalamic–pituitary–adrenal (HPA) axis, begins with signals from the hypothalamus, proceeds through the pituitary gland, and culminates in adrenal hormone release. The HPA axis plays a pivotal role in maintaining hormonal balance and regulating physiological processes; dysregulation, whether overactive or underactive, can lead to dysfunction across multiple organs. Recent research has increasingly focused on the involvement of the HPA axis in the pathophysiology of irritable bowel syndrome (IBS). Studies have indicated that an overactive HPA axis can contribute to IBS, characterized by heightened ACTH production and subsequently elevated cortisol levels in affected individuals [12,13,14,15,16]. Changes in pituitary morphology may underlie this overproduction of ACTH. However, the specific relationship between stress-induced ACTH production and pituitary gland morphology remains largely unexplored. To address this gap, our study aims to investigate whether there is a correlation between IBS and alterations in pituitary gland morphology, given the condition’s association with ACTH overproduction. We assess the pituitary volume and grayscale levels as potential indicators. The use of grayscale analysis has been effective in discerning anatomical differences in conditions like amblyopia, and we apply this methodology to study pituitary gland characteristics in humans [19].

Our study seeks to evaluate the potential of diagnosing IBS by analyzing morphological variations in the pituitary gland using MRI, specifically focusing on pituitary volume and grayscale intensity as key metrics. Additionally, we aim to investigate potential risk factors—such as age, gender, and body mass index (BMI)—that may influence changes in pituitary morphology (volume and grayscale).

## 2. Materials and Methods

### 2.1. Study Design and Subjects

This retrospective chart review was conducted at Zahraa Hospital University Medical Centre (ZHUMC) in Lebanon, and the study received approval from their institutional review board (IRB) under reference number 1/2023. We queried the Picture Archiving and Communication System (PACS) archives from January 2020 to June 2022 to identify all patients (*n* = 60) who underwent an MRI examination of the sella at our institution.

### 2.2. Inclusion and Exclusion Criteria

Exclusion criteria were defined as follows: (1) patients under 18 years of age, (2) patients who underwent MRI sella imaging without contrast-enhanced weighted-T1 sequences, (3) patients with a history of gastrointestinal (GI) disorders other than IBS, including inflammatory bowel disease, celiac disease, lactose intolerance, colorectal cancer, GI infections, diverticular disease, ischemic or mesenteric colitis, fat malabsorption, acute or chronic pancreatitis, and pancreatic cancer, (4) patients with a history of surgical interventions involving the GI tract (such as cholecystectomy, appendectomy, bowel resection, and bariatric surgeries), (5) patients who had undergone pituitary gland surgery, (6) patients taking medications known to cause GI side effects, such as opiates, calcium channel blockers, and antidepressants. Inclusion criteria encompassed all patients older than 18 years who underwent pituitary imaging and did not meet any of the exclusion criteria.

### 2.3. Data Collection

After obtaining their oral consent, eligible subjects who had sufficient MRI sequences were provided with an electronic questionnaire. This questionnaire included sections for demographic information, medical history, surgical history, current medications, and the Birmingham Irritable Bowel Syndrome (IBS) Symptom Questionnaire [20]. The questionnaire consists of 11 items with responses graded on a 6-point scale from 0 (none of the time) to 5 (all of the time). The total score from all items reflects the overall severity of IBS symptoms, with higher scores indicating a greater likelihood of an IBS diagnosis.

### 2.4. Study of the Pituitary Gland and the Pons

For patients meeting our inclusion criteria, we evaluated the pituitary gland in profile for measurements of width, depth, volume, and grayscale intensity. To ensure consistency in our analyses and account for potential confounding factors unrelated to IBS, we also measured these parameters in the pons, a region of the midbrain known not to correlate with IBS. These measurements were used for normalization purposes in our study.

### 2.5. Volume Measurement

**Figure 1:**
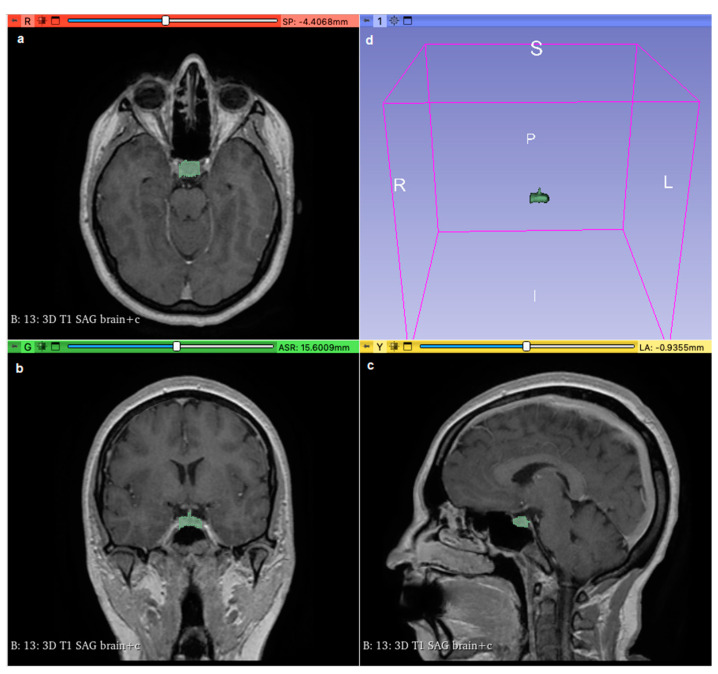
Using 3D Slicer software (version 5.0.2 r30822/a4420c3), the pituitary gland (shown in green area) was delineated and covered all its surface areas on axial (**a**), coronal (**b**), and sagittal (**c**) sections; (**d**) shows the pituitary gland in a 3D view.

Using Three-Dimensional Slicer software (version 5.0.2 r30822/a4420c3), we analyzed contrast-enhanced weighted-T1 sequences from each participant’s MRI to measure the volumes of the pituitary gland and the pons. Under the supervision of a certified radiologist to ensure accuracy, we manually delineated the borders of the pituitary gland using the ‘Segment Editor’ tool, covering its entire surface area (Figure 1). This delineation was performed across all imaging planes (sagittal, coronal, and axial), and the ‘Grow from seeds’ functionality was employed to refine and smooth the selection. Subsequently, ‘Segment Statistics’ provided measurements of pituitary gland volume in cubic millimeters, including voxel counts. The same procedure was applied to delineate and measure the volume of the pons. To facilitate comparative analysis, we calculated the normalized volume of the pituitary gland by dividing its volume by that of the pons.

### 2.6. Grayscale Measurement

**Figure 2:**
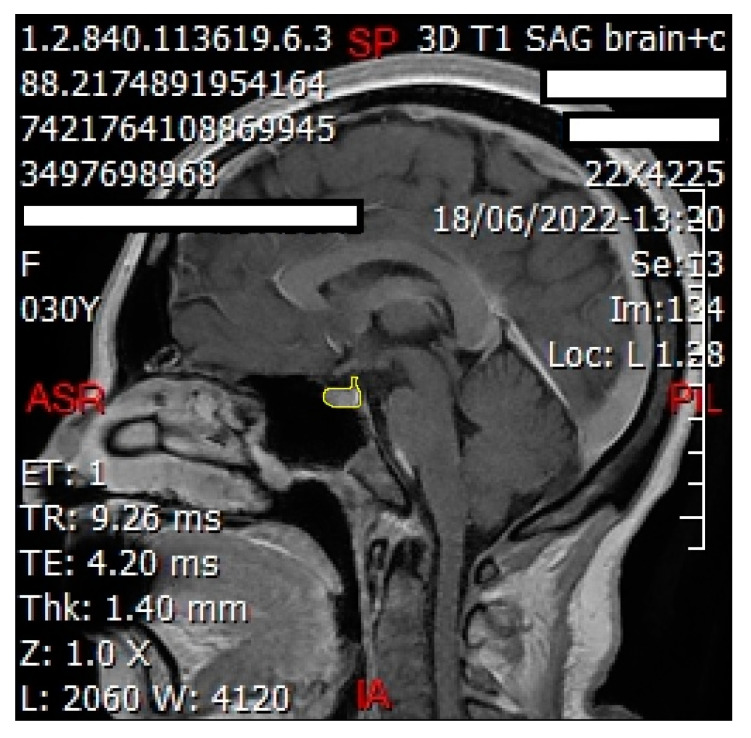
Sagittal cut of the brain showing delineation of the pituitary gland (shown in yellow line) to obtain the grayscale using ImageJ Fiji Software. This technique was performed for each sagittal cut involving the pituitary gland for each patient.

**Figure 3:**
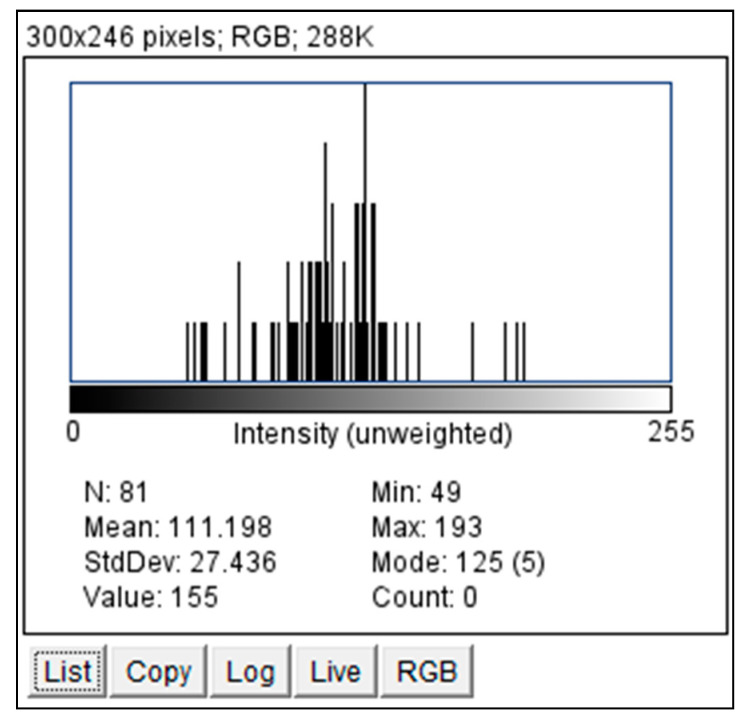
Histogram of the grayscale for the pituitary gland delineated in Figure 2 using ImageJ Fiji Software. The Histogram of grayscale for each cut of the pituitary gland in each patient was obtained by the same technique.

**Figure 4:**
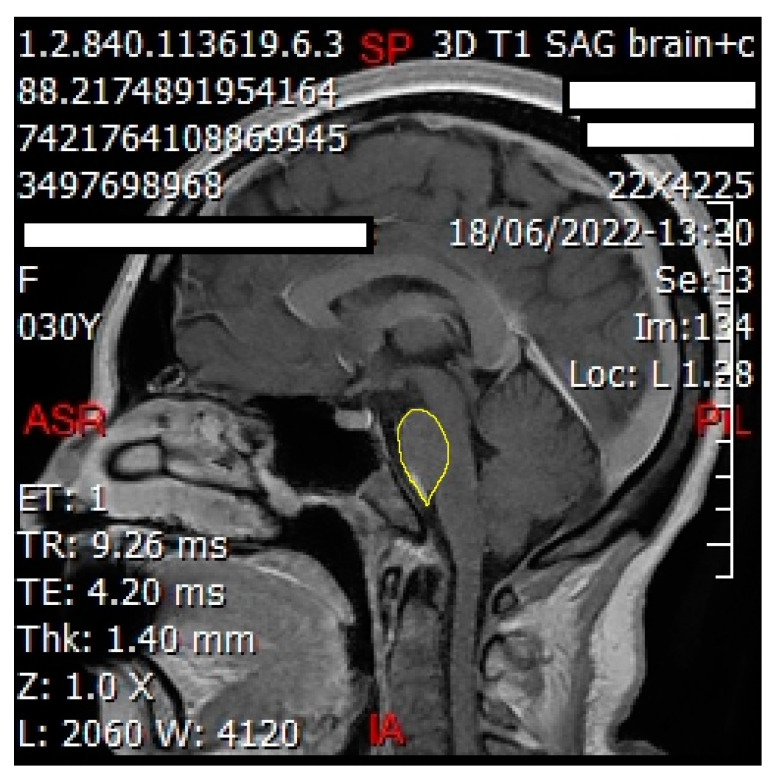
Sagittal cut of the brain showing delineation of the pons (shown in yellow line) to obtain the grayscale using ImageJ Fiji Software. This technique was performed for each sagittal cut involving the pons for each patient.

**Figure 5:**
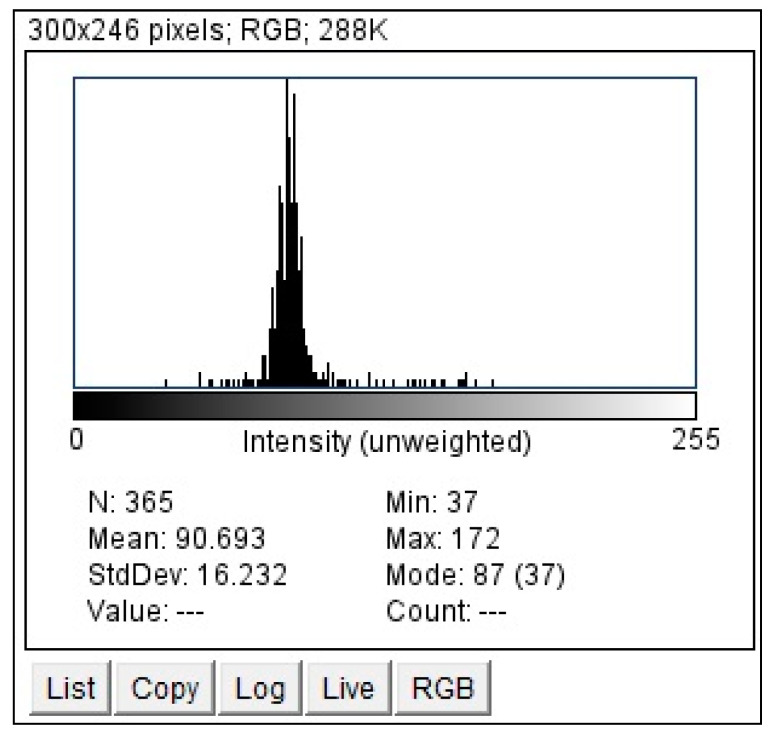
Histogram of the grayscale for the pons delineated in Figure 4 using ImageJ Fiji Software. The Histogram of grayscale for each cut of the pons in each patient was obtained by the same technique.

We employed ImageJ Fiji Software (Version 1.53q) to quantify the grayscale intensities of both the pituitary gland and the pons. After verifying sagittal sections containing the pituitary gland (Figure 2 and Figure 3) with a certified radiologist, we delineated its borders using the ‘Freehand Selections’ tool. Within the ‘Analyze’ tab, histograms were generated for each section, providing mean and standard deviation values of grayscale intensities. Using SPSS 25 (Statistical Package for the Social Sciences, IBM, Armonk, NY, USA), we calculated the overall mean and standard deviation across all sections for both the pituitary gland and the pons (mean and standard deviation from each section). The same methodology was applied to analyze the grayscale intensities of the pons (Figure 4 and Figure 5), ensuring consistency for normalization purposes. The normalized grayscale intensity was determined by dividing the mean grayscale intensity across all sections of the pituitary gland by that of the pons.

### 2.7. Statistical Analyses

Categorical variables were expressed as absolute numbers and percentages. Continuous variables were described either as median and interquartile range or mean and standard deviation, according to their distribution. The t-test or Mann–Whitney test for continuous and the chi-squared or Fisher’s exact test for categorical variables were used to compare pituitary features between patients with IBS and patients without IBS. Data were analyzed using SPSS 25 (IBM, Armonk, NY) and a *p*-value < 0.05 was considered statistically significant.

Bivariate analysis was carried out for comparison of demographic, clinical, and radiologic findings variables across the 2 strata representing those with and without IBS. All variables followed normal distribution except for the variable “volume of the pituitary gland” Shapiro–Wilk test *p* < 0.01. For all continuous variables except for the volume of the pituitary gland, an independent t-test comparing the mean across both strata was performed and both, the mean, and the standard deviation, are shown. For the volume of the pituitary gland, non-parametric tests were used: the Mann–Whitney, Kruskal–Wallis, and Fisher’s exact tests as appropriate. The volume of the pituitary gland variable was presented as median and range. For the categorical variables, a chi-square test was run, and data were represented as frequency percentages. Further analysis was performed using bivariate analysis for the grayscale and the volume of the pituitary gland across gender groups (male and female) and BMI classes (underweight, normal weight, overweight, and obese). A *p*-value less than 0.05 was used to denote statistical significance. All statistics were performed using SPSS 25 (IBM, Armonk, NY).

## 3. Results

Among the 60 MRI scans retrieved from our database, 50 had adequate contrast-enhanced weighted-T1 sequences suitable for further analysis. Following oral consent, these patients were administered the aforementioned electronic questionnaire. A total of 45 subjects responded, resulting in a response rate of 90%. However, seven patients were subsequently excluded from the study: two had undergone pituitary surgeries, one had a history of cholecystectomy, two were using antidepressants, one was taking a calcium channel blocker, and one reported lactose intolerance. Consequently, 38 subjects were ultimately enrolled in the study. Thus, the study comprised 38 participants selected from the initial 60 patients. The mean age of the participants was 36.63 years (±11.01 standard deviation [SD]). Among these, 71.1% (27/38) were female and 28.9% (11/38) were male. Participants were categorized into four BMI groups: 5.3% (2/38) were underweight (BMI < 18.5 kg/m^2^), 26.3% (10/38) were of normal weight (BMI between 18.5 and <25 kg/m^2^), 52.6% (20/38) were overweight (BMI between 25 and <30 kg/m^2^), and 15.8% (6/38) were obese (BMI ≥ 30 kg/m^2^). Among the participants, 55.3% (21/38) were diagnosed with IBS, while 44.7% (17/38) did not meet the criteria for an IBS diagnosis (Table 1).

Among patients diagnosed with IBS, 76.2% were female (16/21) and 23.8% were male (5/21), whereas, among those without IBS, 64.7% were female (11/17) and 35.3% were male (6/17). The majority of patients with IBS were classified as overweight (61.9%, 13/21) or obese (19%, 4/21). The mean age of patients diagnosed with IBS was 38.43 years (±11.61 SD), compared to 34.41 years (±10.11 SD) for non-IBS patients. Statistical analysis indicated no significant differences in the diagnosis of IBS based on age, gender, or BMI (*p* values of 0.27, 0.49, and 0.30, respectively) (Table 2).

The pituitary gland volume for each patient was normalized by dividing it by the volume of the pons, yielding individual normalized volumes. Statistical analysis of the normalized pituitary gland volumes was performed using non-parametric tests due to their non-normal distribution (Shapiro–Wilk test, *p*-value < 0.05). The median normalized volume of the pituitary gland was 0.18 (range: 0.09–0.57) (Table 3). The normalized volume of the pituitary gland relative to the pons showed a positive correlation with the diagnosis of IBS (*p*-value < 0.05). However, there were no significant differences observed about gender or BMI (*p*-values of 0.66 and 0.64, respectively).

Regarding grayscale analysis, each patient’s MRI slices containing the pituitary gland were used to calculate the mean grayscale value. After averaging across all slices, the overall mean grayscale value for the pituitary gland was determined for each participant. The same methodology was applied to calculate the grayscale value of the pons. Subsequently, by dividing the pituitary gland’s grayscale value by that of the pons, we obtained the normalized grayscale value of the pituitary gland relative to the pons. The mean normalized grayscale value of the pituitary gland for all participants in this study was 1.25 (±0.34 SD) (Table 4). Among patients with IBS, the normalized grayscale value of the pituitary gland was 1.19 (±0.32 SD), compared to 1.31 (±0.37 SD) in patients without IBS, with a non-significant *p*-value of 0.31. Similarly, there were no significant differences observed in normalized grayscale values based on gender or BMI of the patients (*p*-values of 0.18 and 0.30, respectively) (Table 4). A comprehensive summary of the study findings is presented in Table 5.

## 4. Discussion

Our study aims to evaluate the potential for diagnosing IBS through morphological alterations in the pituitary gland, specifically changes in volume and grayscale, using MRI as the gold standard technique. Our findings have established a correlation between pituitary gland volume and the presence of IBS. To elucidate this correlation, further investigation into the pathophysiology of IBS was undertaken.

The pathophysiology of IBS remains ambiguous, with various theories proposed [1]. One hypothesis suggests an overactivation of the hypothalamic–pituitary–adrenal (HPA) axis, evidenced by elevated cortisol levels due to increased adrenocorticotropic hormone (ACTH) release from the pituitary gland in individuals with IBS, potentially secondary to elevated corticotropin-releasing factor (CRF) levels [11]. Extensive preclinical research has underscored the role of the CRF-CRF1 receptor signaling system in regulating endocrine, autonomic, behavioral, and visceral responses to stress, suggesting these receptors as potential therapeutic targets for functional bowel disorders [21]. Studies conducted in animal models, such as that of Buckley et al. (2014), have demonstrated that the inhibition of interleukin-6 (IL-6) and CRF1 receptors in vivo normalized stress-induced defecation (*p* < 0.01) and visceral pain sensitivity (*p* < 0.001) in an IBS rat model, indicating a potential therapeutic strategy [22]. Conversely, recent human studies have reported higher cortisol secretion in individuals with IBS compared to healthy controls. For instance, Sagamit et al. (2003) found that intravenous administration of a non-CNS penetrable CRF receptor antagonist attenuated exaggerated motility induced by colonic distention and rectal mucosa stimulation in patients with diarrhea-predominant IBS [23]. Dinan et al. (2006) conducted a study involving 151 subjects, showing that patients with IBS exhibited HPA axis overactivation characterized by elevated cortisol and proinflammatory cytokine levels across all IBS subgroups [12]. Labus et al. (2013) conducted a randomized double-blind study involving women with IBS, suggesting that while CRF signaling through CRF1 receptors plays a role in fear acquisition and extinction learning related to anticipated abdominal pain in both patients and controls, this system appears to be upregulated specifically in patients with IBS [24]. Additionally, a randomized controlled trial in 2014 involving female IBS patients highlighted sustained HPA axis activity following acute psychosocial stress, leading to increased salivary cortisol levels and exacerbation of gastrointestinal symptoms [13,25]. Further studies have corroborated these findings, demonstrating alterations in the HPA axis under baseline and stress conditions using laboratory assessments [14,15,16]. A systematic review by Schaper et al. (2022) summarized findings on the response to acute physiological stress in IBS patients, revealing mixed results that underscore the complexity of understanding IBS pathophysiology. While many studies reported no significant differences in autonomic nervous system measures between groups, psychosocial stressors consistently activated the HPA axis, with varying cortisol responsivity observed across studies [26].

Brain imaging’s role in diagnosing and understanding the pathophysiology of IBS has been explored across various studies focusing on different brain regions. Derbyshire (2003) reviewed the use of functional imaging techniques to investigate painful sensations in IBS patients but found no significant differences compared to controls during visceral distention and somatic sensation experiments [27]. Seminowicz et al. (2010) employed MRI-based voxel-based morphometry and cortical thickness analyses in a well-screened cohort of 55 IBS patients and 48 healthy individuals, revealing gray matter density changes in brain regions associated with cognitive and evaluative functions in IBS patients [28]. Chen et al. (2011) utilized MRI diffusion tensor imaging (DTI) to investigate white matter integrity in 10 female IBS patients compared to 16 healthy female controls, identifying abnormalities in white matter structures within pain-processing regions such as the insula and anterior cingulate cortex (ACC) [29]. In their systematic review, Nistico et al. (2022) synthesized the literature on functional neuroimaging in IBS, highlighting similarities in brain alterations with other functional disorders and emphasizing findings of glutamatergic dysfunction in the anterior insula and dysregulation of the HPA axis in IBS patients [30]. Despite the extensive literature on brain imaging in IBS, no studies were identified investigating pituitary gland size or morphological changes in the exaggerated responses observed. Elevated cortisol levels in patients with IBS may lead to increased ACTH secretion from the pituitary gland, potentially resulting in hypertrophy due to heightened ACTH-producing cell activity. Our study found a positive correlation between pituitary gland volume and the diagnosis of IBS (*p*-value of 0.002, Table 3), indicating that larger pituitary gland volumes are associated with a higher likelihood of IBS diagnosis. Subjects with IBS exhibited larger pituitary glands compared to non-IBS subjects. Importantly, normalized pituitary gland volume did not differ significantly based on gender or BMI of the patients (*p*-values of 0.657 and 0.639, respectively), addressing our secondary objective. Interestingly, gender and BMI did not influence the observed differences in pituitary gland volume (Table 3), ruling out these factors as potential biases contributing to the observed morphological changes in the master gland.

Therefore, this analysis successfully tested and established a correlation between the size of the pituitary gland and the diagnosis of IBS. Previous studies have indicated that IBS is associated with elevated cortisol and ACTH levels, which could explain the observed larger pituitary gland volumes. Our findings confirm this hypothesis. Importantly, potential confounding factors such as female gender and obesity did not influence pituitary gland volume, reaffirming the direct association observed between pituitary gland size and IBS diagnosis in this study.

Patients diagnosed with IBS exhibited a normalized grayscale of 1.19 (±0.32 SD), whereas those without IBS had a grayscale of 1.31 (±0.37 SD) (Table 4). Despite this observed difference, statistical analysis revealed a non-significant *p*-value of 0.312. Similarly, normalized grayscale did not differ significantly based on gender (*p*-value = 0.178) or BMI (*p*-value = 0.296) of the patients. Therefore, our findings indicate that grayscale measurements did not show a significant association with the diagnosis of IBS, nor did they correlate with gender or BMI.

Our study represents the first investigation to directly examine the role of the HPA axis and its implications in the pathophysiology of IBS using pituitary gland imaging. Previous studies have primarily focused on analyzing changes in white and grey matter rather than examining pituitary gland morphology. We have successfully demonstrated that the pituitary gland can exhibit morphological alterations, such as changes in volume, in patients with IBS. This finding aligns with existing literature that supports the involvement of the HPA axis in the pathophysiology of this syndrome. Artificial intelligence (AI) holds great promise in revolutionizing healthcare by enabling advanced diagnostic and therapeutic approaches for various disorders. Our study utilized advanced software technology to quantify glandular changes, suggesting that with larger datasets and machine learning algorithms in future studies, we may unlock new insights into the diagnosis and pathophysiology of IBS through pituitary imaging. A major trend in IBS research has been the move towards incorporating biomarkers and novel diagnostic tools. Traditional diagnostic methods, such as the Rome criteria and exclusion of other conditions, are being complemented by new approaches that aim to identify specific biomarkers associated with IBS. Studies such as those by Goyal et al. (2019) and Chowdhury et al. (2021) have highlighted the potential of biomarkers, including fecal calprotectin and serological markers, to enhance diagnostic accuracy and differentiate IBS from other functional gastrointestinal disorders [31,32]. Researchers are also exploring integrative and personalized medicine approaches to IBS. This involves considering individual patient profiles, including genetic, environmental, and lifestyle factors. For instance, recent research by ElSalhy et al. (2019) underscores the importance of personalized dietary interventions, such as the low FODMAP diet, tailored to individual patient needs. Additionally, there is growing interest in the role of the gut–brain axis and how psychological factors interplay with IBS symptoms [33]. Studies like those by Fernandes et al. (2023) have demonstrated the impact of stress management and psychological therapies on symptom relief, highlighting the need for a holistic approach [34]. Despite these advancements, challenges remain. The variability in IBS symptoms and the lack of definitive diagnostic tests complicate the development of standardized diagnostic and treatment protocols. Furthermore, there is a need for continued research into the pathophysiological mechanisms of IBS to better inform diagnostic criteria and therapeutic approaches. Future research directions may include exploring the role of the microbiome in greater detail, developing non-invasive diagnostic tools, and enhancing patient stratification techniques.

Our study has several limitations that warrant consideration. Firstly, the sample size is small, which limits the generalizability of our findings to the broader population. The online survey method may have contributed to a lower participation rate among older individuals who are less familiar with digital devices. Another noteworthy limitation is the absence of prior studies in the literature addressing similar research questions. Additionally, it is important to note that the software tools ImageJ Fiji and 3D Slicer lack FDA approval for medical diagnostic purposes and are intended solely for experimental and research applications. Moreover, one of the primary limitations of this study is the relatively small sample size of 38 patients. While our findings regarding the morphological changes of the pituitary gland in individuals with irritable bowel syndrome (IBS) provide valuable insights, the limited cohort size poses significant constraints on the generalizability of these results. The small sample size can affect the robustness and external validity of our conclusions. Specifically, it limits our ability to make broad inferences about the relationship between IBS and pituitary gland morphology across diverse populations. The statistical power of the study is also impacted, potentially reducing the sensitivity to detect smaller, but clinically relevant, effects. In light of these limitations, it is crucial to interpret the findings with caution. Future research with larger sample sizes is needed to confirm our results and enhance the generalizability of the observed morphological changes. Additionally, including diverse patient populations and controlling for potential confounding factors could provide a more comprehensive understanding of the relationship between IBS and pituitary gland morphology. We recommend that subsequent studies aim to replicate these findings with larger cohorts and explore the underlying mechanisms further. Several proposed future studies that could validate the findings includes a multicenter longitudinal study, genetic and biomarker study, randomized control trials of IBS treatments, and comparative study of IBS subtypes. This will help establish whether the observed changes are consistent across different patient groups and contribute to a more nuanced understanding of the interplay between IBS and pituitary gland function. On the other hand, the observed high prevalence of IBS symptoms (55.3%) among the study participants raises several important considerations. Firstly, it is crucial to note that the study population was specifically selected from patients who had undergone sellar MRI with contrast at a medical center, likely due to clinical indications unrelated to IBS. These patients may not be representative of the general population in terms of IBS prevalence, as they were not randomly selected but rather were those who had already undergone MRI for other medical reasons. The decision to evaluate these patients for IBS symptoms alongside their MRI assessments could stem from several reasons. First, it may have been an opportunity to opportunistically gather clinical data on a cohort that had already undergone imaging, potentially increasing efficiency and reducing additional burden on patients. Furthermore, there might have been clinical suspicion or anecdotal evidence suggesting a possible link between pituitary gland morphology and functional gastrointestinal disorders, prompting the investigation. However, it is essential to interpret the prevalence of IBS symptoms within this context and not generalize it to the broader population. Studies conducted in clinical settings or with specific patient populations often yield higher prevalence rates than community-based studies due to selection bias and underlying medical conditions prompting medical evaluations. Therefore, while the observed prevalence in this study is notable within its specific cohort, caution should be exercised in extrapolating these findings to the general population’s prevalence of IBS. Furthermore, our study observed a higher prevalence of IBS among females compared to males. This discrepancy may be attributed to our initial sample size, which comprised 27 females and 11 males. We acknowledge that our current study did not separately analyze the results by gender, which could potentially provide deeper insights into how morphological changes in the pituitary gland might differ between male and female IBS patients. Gender-specific analyses could reveal important variations in the relationships between IBS and pituitary morphology, considering the known differences in symptom presentation and hormonal influences between sexes. Lastly, the normalization of pituitary measurements using parameters from the pons is a crucial methodological step in MRI studies, particularly when comparing anatomical structures or volumes across individuals. Without normalization, the raw measurements of the pituitary gland could be influenced by variations in overall brain size or individual anatomical differences, making direct comparisons between patients difficult and potentially misleading. The choice to use the anterior part of the pons for normalization, as depicted in Figure 4 of the study, is likely based on anatomical considerations. The anterior part of the pons is a relatively stable and well-defined anatomical landmark in MRI imaging, making it suitable for consistent measurements across different subjects. Manual marking of this region, when performed accurately by experienced radiologists or researchers using appropriate software tools, ensures that the measurements are precise and reproducible. However, it is essential to acknowledge that manual marking is subject to some degree of intra- and inter-rater variability, which could introduce minor measurement inaccuracies. In summary, normalization using the anterior part of the pons is a methodological approach to enhance the reliability and interpretability of pituitary gland measurements in MRI studies. It minimizes confounding factors related to individual brain anatomy and allows for more meaningful comparisons between subjects. While manual marking of the anterior pons can be accurate when performed meticulously, ensuring consistency and reliability in the measurements is critical for obtaining valid study outcomes.

Despite these limitations, our study successfully validates our initial hypothesis. The novelty of the topic explored in our study lies in its unprecedented focus on correlating morphological changes in the pituitary gland with irritable bowel syndrome (IBS). To our knowledge, no previous studies have investigated this specific relationship using radiological imaging techniques. This pioneering approach fills a significant gap in the existing literature, shedding light on potential anatomical markers associated with IBS pathology that have not been explored before. By identifying a positive correlation between pituitary gland volume and IBS, our research introduces a new avenue for understanding the underlying mechanisms of this complex gastrointestinal disorder. This novel perspective underscores the importance of further research to validate and expand upon our findings, potentially influencing future diagnostic and therapeutic strategies in the management of IBS. The implications of our study into clinical practice are profound, as we have identified a potential anatomical marker—the volume of the pituitary gland—that correlates positively with irritable bowel syndrome (IBS). Moreover, understanding the morphological changes in the pituitary gland associated with IBS may pave the way for targeted treatments that address underlying physiological mechanisms. Further validation through larger studies and exploration of additional morphological features using advanced imaging techniques could enhance the precision of these diagnostic and therapeutic approaches, ultimately improving patient outcomes in clinical settings. Given its exploratory nature, the small sample size can be justified as a preliminary investigation to establish an association for future larger-scale studies. Lastly, our study employed a validated questionnaire for the diagnosis of IBS, enhancing the reliability of our findings.

## 5. Conclusions

In conclusion, our study successfully identified a positive correlation between IBS and changes in the pituitary gland, specifically in terms of pituitary gland volume, although no correlation was found concerning the pituitary gland grayscale. This research represents the first attempt to establish a radiological correlation between pituitary gland morphology and IBS using computer software. Given our small sample size and the absence of prior similar studies in the literature, larger sample sizes are warranted to enhance statistical power and enable more robust analysis. Subsequent investigations could delve deeper into the pathophysiological mechanisms underlying this correlation, explore potential treatment modalities, and investigate the potential role of artificial intelligence in the diagnostic assessment of such pathologies.

## Figures and Tables

**Table 1:** Socio-demographic characteristics of the study participants (*n* = 38) including age expressed as mean ± standard deviation. Gender, BMI, and IBS diagnosis are represented as frequencies and percentages.

Variable		Mean ± SD
**Age (years)**		36.63 ± 11.01
		**(*n*) %**
**Gender**	Female	27 (71.1)
	Male	11 (28.9)
**BMI (Kg/m^2^)**	Underweight	2 (5.3)
	Normal	10 (26.3)
	Overweight	20 (52.6)
	Obese	6 (15.8)
**IBS**	Positive	21 (55.3)
	Negative	17 (44.7)

**Table 2:** Socio-demographic factors in a sample of Lebanese adults (*n* = 38) by diagnosis of IBS.

Variable		IBS Patients (*n*) %	Non-IBS Patients (*n*)%	*p*-Value
**Gender**	Female	16 (76.2)	11 (64.7)	0.491
	Male	5 (23.8)	6 (35.3)	
**BMI (Kg/m^2^)**	Underweight	1 (4.8)	1 (5.9)	0.303 *
	Normal	3 (14.3)	7 (41.2)	
	Overweight	13 (61.9)	7 (41.2)	
	Obese	4 (19)	2 (11.8)	

* Fisher’s exact; significant *p*-value < 0.05.

**Table 3:** The median normalized volume of the pituitary gland for all participants (*n* = 38) expressed as median and interquartile range. The mean and standard deviation for the normalized volume of the pituitary gland were represented according to gender, BMI, and IBS diagnosis.

Variable		Median [IQR]	*p*-Value
**Normalized volume for all patients (*n* = 38)**		0.18 [0.09–0.57]	
**Normalized volume**		**Mean ± SD**	
**Gender**	Female	0.19 ± 0.09	0.657 *
	Male	0.21 ± 0.12	
**BMI (Kg/m^2^)**	Underweight	1.17 ± 0.26	0.639 **
	Normal	1.36 ± 0.41	
	Overweight	1.26 ± 0.30	
	Obese	1.02 ± 0.36	
**IBS**	Positive	0.22 ± 0.09	0.002 *
	Negative	0.17 ± 0.11	

* Mann–Whitney; ** Kruskal–Wallis; significant *p*-value < 0.05.

**Table 4:** The median normalized grayscale of the pituitary gland for all participants (*n* = 38) and then distributed according to gender, BMI, and diagnosis of IBS.

Variable		Mean ± SD	*p*-Value
**Normalized grayscale for all patients (*n* = 38)**		1.25 ± 0.34	
**Gender**	Female	1.29 ± 0.32	0.178 *
	Male	1.31 ± 0.39	
**BMI (Kg/m^2^)**	Underweight	1.17 ± 0.26	0.296 **
	Normal	1.36 ± 0.41	
	Overweight	1.26 ± 0.30	
	Obese	1.02 ± 0.36	
**IBS**	Positive	1.19 ± 0.32	0.312 *
	Negative	1.31 ± 0.37	

* *t*-test for independent samples; ** one-way ANOVA; significant *p*-value < 0.05.

**Table 5:** Comprehensive summary of the study findings.

Parameters	Patients with IBS (*n* = 21)	Patients without IBS (*n* = 17)	*p*-Value
Gender			
Female	76.2% (16/21)	64.7% (11/17)	
Male	23.8% (5/21)	35.3% (6/17)	0.49
BMI (Kg/m^2^)			
Underweight (%)	5.3% (1/21)	5.9% (1/17)	
Normal weight (%)	26.3% (5/21)	29.4% (5/17)	
Overweight (%)	61.9% (13/21)	47.1% (8/17)	
Obese (%)	19.0% (4/21)	17.6% (3/17)	0.30
Age (years)			
Mean ± SD	38.43 ± 11.61	34.41 ± 10.11	0.27
Pituitary Volume (mm^3^)			
Median (IQR)	180 (90–570)	150 (80–390)	0.002
Normalized Grayscale			
Mean ± SD	1.19 ± 0.32	1.31 ± 0.37	0.312

## Data Availability

The data presented in this study are available on request from the corresponding author. The data are not publicly available due to patients’ privacy; they were only used for this study and will not be made freely accessible to anyone.
